# Damage control resuscitation: how it’s done and where we can improve. A view of the Brazilian reality according to trauma professionals

**DOI:** 10.1590/0100-6991e-20243785-en

**Published:** 2024-12-02

**Authors:** MARCELO AUGUSTO FONTENELLE RIBEIRO, LETICIA STEFANI PACHECO, JUAN CARLOS DUCHESNE, JOSE GUSTAVO PARREIRA, SHAHIN MOHSENI

**Affiliations:** 1 - University of Maryland, R Adams Cowley Shock Trauma Center - Baltimore - MD - Estados Unidos; 2 - Pontifical Catholic University of São Paulo - Campus Sorocaba, Discipline of Trauma Surgery - Sorocaba - SP - Brasil; 3 - Tulane University School of Medicine, Division Trauma, Acute Care & Critical Care Surgery - New Orleans - LA - Estados Unidos; 4 - Santa Casa School of Medical Sciences, Department of Surgery - São Paulo - SP - Brasil; 5 - School of Medical Sciences Orebro university, Department of Surgery - Orebro - OR - Suécia

**Keywords:** Hemorrhage, Resuscitation, Hypotension, Blood Transfusion, Shock, Hemorrhagic, Hemorragia, Ressuscitação, Hipotensão, Transfusão de Sangue, Choque Hemorrágico

## Abstract

**Introduction:**

Hemorrhage is the leading cause of preventable deaths in trauma patients, resulting in 1.5 million deaths annually worldwide. Traditional trauma assessment follows the ABC (airway, breathing, circulation) sequence; evidence suggests the CAB (circulation, airway, breathing) approach to maintain perfusion and prevent hypotension. Damage Control Resuscitation (DCR), derived from military protocols, focuses on early hemorrhage control and volume replacement to combat the “diamond of death” (hypothermia, hypocalcemia, acidosis, coagulopathy). This study evaluates the implementation of DCR protocols in Brazilian trauma centers, hypothesizing sub-optimal resuscitation due to high costs of necessary materials and equipment.

**Methods:**

In 2024, an electronic survey was conducted among Brazilian Trauma Society members to assess DCR practices. The survey, completed by 121 participants, included demographic data and expertise in DCR.

**Results:**

All 27 Brazilian states were represented in the study. Of the respondents, 47.9% reported the availability of Massive Transfusion Protocol (MTP) at their hospitals, and only 18.2% utilized whole blood. Permissive hypotension was practiced by 84.3%, except in traumatic brain injury cases. The use of tranexamic acid was high (96.7%), but TEG/ROTEM was used by only 5%. For hemorrhage control, tourniquets and resuscitative thoracotomy were commonly available, but REBOA was rarely accessible (0.8%).

**Conclusion:**

Among the centers represented herein, the results highlight several inconsistencies in DCR and MTP implementation across Brazilian trauma centers, primarily due to resource constraints. The findings suggest a need for improved infrastructure and adherence to updated protocols to enhance trauma care and patient outcomes.

## RESUMO

**Introdução:**

A hemorragia é a principal causa de morte evitável em vítimas de trauma, resultando em 1,5 milhões de mortes por ano em todo o mundo. A Reanimação durante o Controle de Danos (RCD), derivada de protocolos militares, centra-se no controle precoce da hemorragia e na reposição de volume para combater o “diamante da morte” (hipotermia, hipocalcemia, acidose, coagulopatia). Este estudo avaliou a utilização de protocolos de RCD por profissionais da área brasileiros, levantando a hipótese de ressuscitação subótima devido aos altos custos dos materiais e equipamentos necessários.

**Métodos:**

Em 2024, foi realizada uma pesquisa eletrônica entre os membros da Sociedade Brasileira de Trauma para avaliar as práticas de RDC. A pesquisa, respondida por 121 participantes, incluiu dados demográficos e experiência em RCP.

**Resultados:**

Todos os 27 estados brasileiros foram representados. Dos entrevistados, 47,9% relataram a disponibilidade do Protocolo de Transfusão Maciça (PTM), e apenas 18,2% utilizaram sangue total. A hipotensão permissiva foi praticada por 84,3%, exceto nos casos de traumatismo cranioencefálico. O uso de ácido tranexâmico foi elevado (96,7%), mas o TEG/ROTEM foi utilizado por apenas 5%. Para o controlo da hemorragia, torniquetes e toracotomia de reanimação foram comuns, mas o REBOA foi raramente acessível (0,8%).

**Conclusões:**

os resultados destacam várias inconsistências na implementação das RCD e MTP, principalmente devido a restrições de recursos. Os achados sugerem a necessidade de melhorar a infraestrutura e a adesão a protocolos atualizados para melhorar o atendimento ao trauma e os resultados dos pacientes.

## INTRODUCTION

Trauma continues to be a leading cause of death, with hemorrhage being the primary cause of preventable fatalities. Annually, trauma-related hemorrhage results in approximately 1.5 million deaths worldwide^1^ ^-^ ^4^. 

Traditionally, the severely injured patient has been evaluated followed by prompt intervention of a sequence: airway, breathing, circulation (ABC). With mounting evidence, this sequence of evaluation has been favoring the “circulation first”, i.e. the circulation-airway-breathing (CAB) approach. This alteration pursues to reassure a maintained perfusion, which alter the overall outcome by avoiding deleterious effects of hypotension on end-organs. 

The primary trauma assessment in the emergency room (ER) focuses on a series of actions aimed at stopping hemorrhage as early as possible as well as replacing loss of intravascular volume^5^. Damage control resuscitation (DCR), derived from military experience, standardizes a combination of measures for severely injured patient in hemorrhagic shock to restore volume and perfusion and to reverse the “diamond of death” (hypothermia, hypocalcemia, acidosis, and coagulopathy). These actions have been shown to reduce mortality^7^ ^,^ ^8^. 

DCR consists of several important domains: recognition of the hemodynamically compromised patient, early control of bleeding source, replacement therapy. For these steps a definitions, techniques and devices have been developed.

The patients indicated to receive DCR measures include those with: hypotension (defined as systolic blood pressure [SBP] of <90mmHg and <110 for ages older than 65 years), shock index (HR/SBP) more than 1 or relation HR>SBP in adults, End-Tidal CO2 <26-28.5mmHg, pelvic fracture, positive FAST, abnormal lactate, abnormal base deficit, altered point-of-care INR and rapid-TEG^7^. Based on the indications above mentioned it is recommended to start with the volume restoration a rapid sequence intubation with lower doses of sedatives drugs to prevent cardiovascular collapse^6^ ^-^ ^9^. 

Considering the importance of carrying out a DCR according to the most up-to-date protocols using the resources needed for adequate resuscitation, this study aims to evaluate the way trauma centers in Brazil carry out these procedures, with the hypothesis that the protocols currently used offer sub-optimal resuscitation due to the costs of obtaining the materials and equipment needed for adequate resuscitation.

## METHODS

This study was a cross-sectional study conducted between April and June of 2024, using an electronic form utilizing the Brazilian Trauma Society mailing list and their WhatsApp group. The questions were based on a recent guideline published by LaGrone et al. in 20247. All obtained responses were utilized for the analysis of the data. The majority of the members are general and trauma surgeons, but some members are also intensivist doctors (03), anesthetists (01), and doctors from other specialties (22) who answer about how DCR is conducted in their institutions. The questionnaire was applied anonymously and voluntarily; the complete survey can be accessed at https://forms.gle/FGcLFKPWfmJfYgDZ8. The questions asked can be separated into two main domains: the demographic profile of the surgeon (Table 1) and expertise on this subject (Table 2). All the results were exposed utilizing the percentage of responses obtained, considering that the study aimed to describe the reality in Brazilian Trauma Centers and not to compare our findings to the literature. 

**Table 1:** Demographic profile questions included in the survey.

1	Name (optional)
2	State where you work
3	Medical qualification (Board certification)
4	Time of experience after training
5	The kind of hospital where the Surgeon works

**Table 2:** Expertise in DCR - the detailed questions are available through the link.

1	Do you have Massive Transfusion Protocol (MTP) at your institution?
2	What criteria do you currently use to activate MTP?
3	Do you practice C-ABCDE at your institution?
4	What do you use as a line to infuse volume in your trauma patients?
5	What kind of volume replacement therapy do you have available at your institution?
6	How you manage blood transfusion in trauma patients?
7	Do you use permissive hypotension for your patients?
8	What kind of adjuncts do you have available to trauma patients related to MTP?
9	What do you have available to stop the bleed in the trauma bay?
10	Indications for ED thoracotomy
11	Which is your first line option to bleeding control in pelvic trauma
12	Do you measure routinely Intra-abdominal pressure in trauma patients?
13	How do you do it?
14	Which of marker do you use to evaluate the physiological response of your patients?

## RESULTS

There were 121 responders, most active members of the Brazilian Trauma Society (100%), and the majority were active general/trauma surgeons (81,8% and 46,3%, respectively). Most of the responders (24,8% of the participants) were practicing in São Paulo, Brazil. Regarding the board certifications from the doctors who answered the survey, 81,8% were board certified general surgeons, and out of them, 46% also sustained a board certification in trauma surgery by the Brazilian Medical Association. In addition, 33,1% were specialized for more than 20 years. Table 3.

**Table 3:** Type of training for the doctors.

Type of training	Total number (%)
General Surgeon	100 (82%)
Trauma Surgeon	57 (46,7%)
Internal Medicine	3 (2,5%)
Anesthesia	2 (1,6%)
Others	27 (22,1%)

*consider overlapping of training, specially trauma surgeons that are also general surgeons

**Figure 1:**
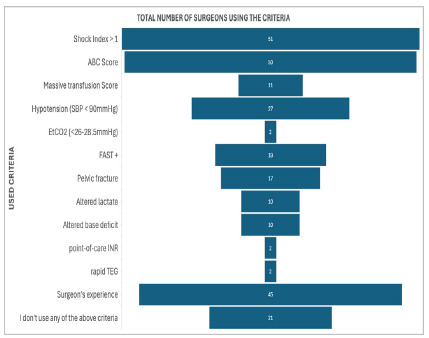
Criteria for Massive Transfusion Protocol activation.

When asked if MTP was available in their hospital, 47,9% responded that it was available, representing less than half of the surgeons. The criteria for activation of the massive transfusion protocol are shown in Figure 1. Additionally, when questioned about prioritizing circulation first, 7,4% answered that they don’t agree with that notion nor practice it on regular basis. Pertaining to securing venous access, most responders used a peripheral intravenous catheters and venous access guided by ultrasound, used in 89,3% and in 75,2% of cases, respectively. 

[Fig f2]

**Figure 2 f2:**
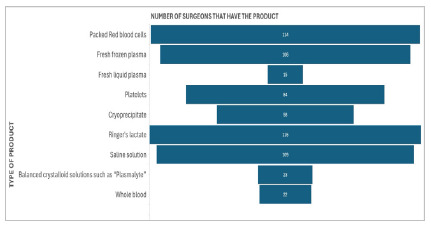
Main options for Volume restoration available.

Other interventions used were the administration of tranexamic acid (96,7%), followed by calcium replacement (71,1%), use of vasopressin in the presence of traumatic brain injury (64,5%), offering heat blankets (63,6%) and doing an arterial blood gas in the ER (50,4%). The use of TEG/ROTEM was only used routinely by 5% of responders. In relation to controlling bleeding lesions, what is most available in the trauma unit is equipment for resuscitative thoracotomy (71,9%), tourniquets (67,8%) and ultrasound exclusively for trauma (66,9%). 

Regarding resuscitative thoracotomy, it was questioned about the signs of life as indications for this procedure and in which situations should it be performed. The signs of life presented were: pupillary response, spontaneous ventilation, presence of the carotid pulse, measurable or palpable arterial pressure, extremities movements or myocardium electrical activity. The situations enlisted were blunt thoracic trauma, penetrating thoracic trauma, and penetrating extra-thoracic trauma, with or without signs of life. All the indicated circumstances were chosen when in the presence of signs of life, with penetrating thoracic trauma being the most designated situation by 78,5%, and the least were blunt thoracic and penetrating extra-thoracic traumas, with 10,7% respectively. 

Regarding the use of REBOA in Brazil it is not available up to the moment although one response was obtained as having it available. 

For pelvic trauma in a hypothetical situation if all the tools are available to bleeding control, as first option to control hemorrhage the majority chose pre peritoneal packing, in 60,3% of the answers, following the use of REBOA in 35,5% and thoracotomy with aortic clamping in 4,1% of the cases. When asked if intra-abdominal pressure was measured regularly, 72,7% don’t practice this, and for those who execute this procedure it is done manually by a three-way catheter. Finally, to evaluate the physiological response to the damage control resuscitation, the most common parameters were represented by dosage of lactate (81,8%), diuresis (77,7%), arterial pressure and pulse (66,1%) and base deficit (57,9%).

## DISCUSSION

In recent decades, the implementation and adherence to Damage Control Resuscitation (DCR) has shown a significant reduction in adverse outcomes and increased survival in hemorrhagic trauma patients^7^.

As a result, by what is shown as results in this research, there are still many inconsistencies about the DCR and MTP techniques, which can lead to worse outcomes in multiple traumatized patients. Thus, we here compare our data to the recent evidential support that guides this approach. Despite the increasing number of hospitals claiming trauma center capabilities, most of these centers lack a well-organized Massive Transfusion Protocol (MTP) and are unable to provide adequate resuscitation for severely injured patients. Essential tools such as warming devices, rapid infusion systems, and blood transfusion protocols often need to be included. Most of the represented hospitals in our sample were part of the public healthcare system (77%). In the United States of America according to Velopulos et al. in 2013 the total cost for trauma patients was 32 billion dollars while in the same period in Brazil the total cost for trauma was around 92 million dollars. The restrictive budget of the public system does not prioritize trauma care, leading to insufficient allocation of necessary resources^10^.

Two recent systematic reviews as well as other studies were able to determine significant mortality benefits related with hypotensive resuscitation, revealing reduced postoperative coagulopathy and short-term overall mortality and improved mortality for blunt trauma patients with the lower SBP goal^7^ ^,^ ^11^ ^,^ ^13^ ^-^ ^17^. Our data demonstrate that the concept of permissive hypotension is well-established among the surgeons who answered our questionnaire. Approximately 84% of surgeons regularly apply permissive hypotension in trauma patients without associated traumatic brain injury (TBI), while 11% reported applying this concept to all patients without exception, which represents a deviation from the current standards. Current evidence indicates that patients with TBI should not be subjected to permissive hypotension, as it is essential to ensure adequate brain perfusion during resuscitation to reduce hypoxia-related injuries. This result characterizes a very selective and specialized population. 

To provide the most effective volume replacement massive transfusion protocol (MTP) will help decreases mortality in trauma by properly supplying blood transfusion or blood products, such as platelets, plasma and blood cells, in 1:1:1 proportion, being preferable whole blood (WB) transfusions to reinstate volume. Nowadays it is well stablished that a minimum administration of crystalloids should be used when blood components are not available, recognizing that it can be prejudicial if given in high quantities, improving mortality. 

The use of crystalloids in trauma has been traditionally put in practice because of the low cost and easy logistics both in prehospital and in-hospital trauma care^18^. The administration of crystalloids to restore volume in trauma has been questioned in the two decades for being associated with a greater incidence of complications, when done in large volumes, such as bowel and retroperitoneal edema, associated with abdominal compartment syndrome (ACS), acute respiratory distress, intensified bleeding due to clot disturbance, worsening of the coagulopathy established by over dilution of the plasma, electrolyte disruption, end-organ dysfunction and increased mortality^3^ ^,^ ^12^ ^,^ ^13^. The PROPPR randomized clinical trial conducted by Holcomb et al in 2015 have shown that for each added 500mL of crystalloid given in the first 6 hours in-hospital resuscitation was related with a 9% increase in acute respiratory distress syndrome, although the number of blood products received was not predictive of acute respiratory distress syndrome^19^. The latest Tactical Combat Casualty Care guidelines for fluid resuscitation does not recommend crystalloids for fluid resuscitation^20^.

When faced with the decision of which crystalloid solution to use for the opening 24 hours of volume restoration, the use of balanced crystalloid solutions, such as Ringer’s solution and Plasmalyte, when compared to normal saline in multiple traumatized patients has been associated with a better acid-base state and decreased hyperchloremia, being also preferred in case of traumatic brain injury^7^ ^,^ ^21^ ^,^ ^22^. In prehospital management, the use of balanced administration of crystalloids is still a viable choice and ought to be initiated in low doses, preferably in 250cc boluses^13^. This research demonstrates that a considerable number of the represented hospitals in this study up to the present time uses crystalloids solution to restore volume, which contrast to what was found on literature. Although we cannot assure that crystalloids were the initial first line of treatment, considering its price, we assume that it still represents a common practice, especially in hospitals where MTP is unavailable. 

In Brazil, up to the moment, there are no specific regulations for the use of whole blood. Unfortunately, we found that based on the answers obtained, more than 50% of the hospitals taking trauma patients do not have an established Massive Transfusion Protocol. With that, most of the trauma patients end up receiving an inadequate volume reposition and develop trauma coagulopathy. Nowadays, very few centers are able to establish an MTP using whole blood (18%); the lack of official regulations regarding blood transfusion for trauma patients creates a complex situation for the trauma centers not only to implement the protocols formally but also to be able to audit the results after the activations. Pursuing the CAB approach, it is reasonable that exsanguinating lesions are assessed promptly since it is a primary source of preventable early death. The implementation of MTP greatly improved the management of trauma patients when treating acute hemorrhage, trauma-induced coagulopathy (TIC), and hemostatic defects, also limiting the use of crystalloids^6^ ^,^ ^7^. To reinstate the lost fluid, the most adequate measure is to replace blood with blood. Volume restoration in trauma care was usually done by blood components at a 1:1:1 ratio of platelets, red blood cells and plasma^14^. Nowadays, it is known that the use of reconstituted whole blood (WB) is preferable, due to the significant reduction in mortality, enhancing the outcomes of trauma patients with exsanguinating injury, especially when is done as early as possible. A study conducted by Brill et al.^23^ has demonstrated that compared to the utilization of blood components versus whole blood transfusion has improved 30-day survival by 60% and has decreased the requirement for 24-hour blood products by 7%, allowing euvolemic resuscitation as “like for like” setting. The benefit of this action is explained by the fact that whole blood reconstitution inhibits TIC^6^. 

This coagulopathy happens due to the depletion of clotting factors by massive hemorrhage, the reduction of efficacy of clotting factors and platelets by hypothermia and hemodilution, hypoperfusion and shock, which leads to acidosis and damages clot formation, associated with the late lethal triad of trauma3. The use of WB is well established by the recent literature. The data from a study conducted by the American College of Surgeons Trauma Quality Improvement Program proved that whole blood combined with component therapy was associated with improved survival (n=2785), compared with component therapy^24^ ^,^ ^25^. Also, a prospective observational study run in fourteen trauma centers (n=1623) reported a significant reduction in 24-hour mortality (14 vs. 32%) and a 48% reduction in hospital mortality^26^. In a cohort study, severe injured patients in need of MTP were related with a 37% and 47% reduced risk of in-hospital mortality at 24 hours and 30 days, respectively^27^. In addition, the Joint Trauma System consensus too defends the use of WB for managing hypovolemic shock^28^. 

The time to give WB to the traumatized patient must be a priority. In a retrospective cohort study by Torres et al, WB given within the first 24 hours of emergency department arrival, at any given point, to patients with massive bleeding injury as early as possible was associated with improved survival, consistently showing that improved outcomes are time-dependent^29^ ^,^ ^30^. 

When in lack of WB, the balanced use of blood components is still a viable choice. In a trial of Holcomb et al, the Pragmatic, Randomized Optimal Platelet and Plasma Ratio (PROPPR), it was shown that severe traumatized patients presented with major bleeding receiving early plasma, platelets, and red blood cells in a 1:1:1 ratio compared to 1:1:2 did not alter significantly 24 hour or 30-day mortality. Although, more patients in the 1:1:1 group achieved hemostasis and fewer deaths were experienced due to exsanguination by 24 hours. The time of initiation of MTP was also studied, and for each 1-minute delay on the protocol application, there was a 5% increase in mortality. The early administration of balanced blood components is associated with less need for more blood components transfusions^7^ ^,^ ^19^ ^,^ ^31^. 

Ultimately, according to priority of use in trauma resuscitation, whole blood, blood components at 1:1:1 ratio, red blood cells and plasma at 1:1 ratio, plasma with or without red blood cells and red blood cells isolated is the order to be followed^32^. 

Using WB, whilst advantageous, still is not widely disseminated in Brazilian trauma centers, attributable to the absence of infrastructure and equipment to acquire and maintain reconstituted whole blood, justifying the considerable number of surgeons in our study to use balanced blood components and other alternatives yet.

Another point to consider are the adjunct measures to be taken during trauma resuscitation. Selective hypotensive resuscitation (SBP goal >70mmHg or MAP goal >50mmHg), not indicated in case of traumatic brain injury^7^. Some other concerns are important take part in post-trauma care, including: preventing and managing hypothermia (temperature <36°C), reverse anticoagulation, correcting acidosis, treating hypocalcemia, correcting trauma-induced coagulopathy (TIC), administering tranexamic acid and low-dose vasopressin when indicated^7^. 

Hypothermia is related to increased need of blood transfusions and mortality, and is caused by exposure to the environment, hemorrhagic shock and the use of cold fluids in volume restoration. The prognosis is directly linked to the gravity of the hypothermia, achieving 100% mortality when the body temperature drops to under 33 degrees Celsius. When preventing hypothermia, it is important to rewarm the patient, preferably the torso than the extremities, by using warm blankets, increasing the room temperature, use of heaters and warm fluids. The extracorporeal warming techniques can raise body temperature at a rate of 4-5°C per hour. To monitor body temperature, the measurement of sublingual, tympanic, or urinary bladder catheter temperature is advocated. The evidence-based goal to start rewarming trauma patients is indicated when body temperature is under 37 degrees Celsius^7^ ^,^ ^33^ ^,^ ^34^. To avoid hypothermia, the most utilized method used by surgeons in the current study was, by availability, warm blankets (63,6%). Devices like rapid infusion systems we reported to be used in Brazil by only 10% of the surgeons, volume warmers in only 19% clearly demonstrating that the cost of this equipment as well as its maintenance still represent a major obstacle for public services, making thermal control as well as rapid volume infusion drastically compromised for critically ill patients.

The reposition of calcium plays an important role in trauma care, as hypocalcemia became the fourth element in the lethal diamond of death. A study stipulated that about 55% of the traumatized patients are presented with hypocalcemia, being aggravated by transfusion of blood and its components, due to the utilization of citrate to preserve blood, which is a calcium chelator^11^. Because hypocalcemia is linked to coagulopathy, worsen acidosis, increased need for transfusions and elevated mortality, some military guidelines suggests maintaining up to 1 to 1.2mmol/L, by giving 1g of calcium, such as calcium gluconate, within the first unit of blood product, and at least after 4 units^5^ ^,^ ^7^ ^,^ ^35^ ^,^ ^36^. For our own part, repositioning of calcium is done by 71,1% of the surgeons involved.

The use of Tranexamic Acid (TXA) is well known to be used in trauma patients, acting as an antifibrinolytic agent, which operates as a lysine receptor antagonist on plasminogen to block fibrinolysis. The administration of TXA within the first 3 hours post trauma injury is associated with reduced mortality, as it reduces bleeding and was proved to be safe even when accompanied by traumatic brain injury^7^ ^,^ ^38^ ^,^ ^39^. The dose recommended is 1-g TXA bolus within 3 hours from injury followed by another 1-g infusion of TXA over 8 hours18. Is stated that the use of TXA in traumatized patients is done in 96,7% of the surgeons in our study.

To evaluate coagulation, Thromboelastography and Rotational Thromboelastometry (TEG/ROTEM) has been defended throughout recent literature. TEG/ROTEM-guided resuscitation can be beneficial, according to a study conducted by Brill et al.^38^ The use of TEG and ROTEM in Brazil is limited due to the high cost of the material, only 5% of the surgeons reported to use regularly this kind of equipment to provide the patients a target direct therapy, with that been said we can assume based on the information obtained by the surgeons answering the questionary that the management of trauma coagulopathy for trauma patients in Brazil remains in a suboptimal condition in most of their institutions.

Another issue is to reverse anticoagulation in those patients who present this condition before trauma, for example when taking warfarin. For these patients, the use of prothrombin complex concentrate has shown efficient results, including with vitamin K antagonists^39^ ^-^ ^41^. The benefit from reverse anticoagulation is still not dependable^7^. Besides the evidential support, in our case prothrombin complex is only used in 12,4% of all participants in the research. 

For hemorrhage control, compressible techniques include tourniquets, pelvic binder and hemostatic dressings are commonly used, lessening hypovolemic shock and enhancing survivability^7^ ^,^ ^9^ ^,^ ^42^. Whereas non-compressible approaches, such as resuscitative endovascular balloon occlusion of the aorta (REBOA), which is a high standard method permitting fast control of the source of bleeding, when used correctly, and is recommended when is deployed in less than 15 minutes for zone 1 lesions and less than 30 minutes for zone 343. When comparing to resuscitative thoracotomy, a 2022 retrospective study conducted by the AAST REBOA study group by Bini et al established better outcomes when used REBOA in pelvic trauma^44^. 

The aim of resuscitative thoracotomy is to control abdominal and pelvic exsanguinating bleeding by supradiaphragmatic aortic clamping and is indicated when in the presence of signs of life^6^. In our study, due to the lack of ample diffusion of REBOA in Brazilian trauma centers, other techniques like the resuscitative thoracotomy and pre peritoneal packing are preferred, considering also the lower costs. Another alternative could be angioembolization, although it is also not available in most of the public hospitals in Brazil. 

Surveys are a valuable tool for collecting data in research, offering a cost-effective and scalable way to gather information from a large and diverse population. Surveys allow for both quantitative and qualitative insights, making them versatile for various types of research. However, their limitations include potential biases, such as selection bias or response bias, where participants may not represent the broader population or may give inaccurate or socially desirable answers. Additionally, the design of survey questions can significantly impact the validity of the data. Despite these limitations, surveys remain a powerful method when carefully designed and used with other research methodologies. In the present study, all Brazilian States were represented, and the data was obtained from a very active group of professionals involved in caring for trauma patients. Therefore, we can assume that the answers reflect the reality in most of the hospitals currently dealing with trauma patients in the country. 

## CONCLUSION

Most of the surgeons interviewed did not follow the most up-to-date guidelines for damage control resuscitation proposed by the American Association for the Study of Trauma / American College of Surgeons. In short, this could be explained by equipment shortfall and possibly the high costs to offer all the required tools for an adequate resuscitation, especially in public hospitals. 
